# Telomere shortening reflecting physical aging is associated with cognitive decline and dementia conversion in mild cognitive impairment due to Alzheimer’s disease

**DOI:** 10.18632/aging.102893

**Published:** 2020-03-03

**Authors:** Seong-Ho Koh, Seong Hye Choi, Jee Hyang Jeong, Jae-Won Jang, Kyung Won Park, Eun-Joo Kim, Hee Jin Kim, Jin Yong Hong, Soo Jin Yoon, Bora Yoon, Ju-Hee Kang, Jong-Min Lee, Hyun-Hee Park, Jungsoon Ha, Young Ju Suh, Suyeon Kang

**Affiliations:** 1 Department of Neurology, Hanyang University College of Medicine, Guri 11923, Korea; 2 Department of Neurology, Inha University School of Medicine, Incheon 22332, Korea; 3 Department of Neurology, Ewha Womans University School of Medicine, Seoul 07985, Korea; 4 Department of Neurology, Kangwon National University School of Medicine, Chuncheon 24289, Korea; 5 Department of Neurology, Dong-A Medical Center, Dong-A University College of Medicine, Busan 49201, Korea; 6 Department of Neurology, Pusan National University Hospital, Pusan National University School of Medicine and Medical Research Institute, Busan 49241, Korea; 7 Department of Neurology, Samsung Medical Center, Sungkyunkwan University School of Medicine, Seoul 06351, Korea; 8 Department of Neurology, Yonsei University Wonju College of Medicine, Wonju 26426, Korea; 9 Department of Neurology, Eulji University Hospital, Eulji University School of Medicine, Daejeon 35233, Korea; 10 Department of Neurology, Konyang University College of Medicine, Daejeon 35365, Korea; 11 Department of Pharmacology, Inha University School of Medicine, Incheon 22212, Korea; 12 Department of Biomedical Engineering, Hanyang University, Seoul 04763, Korea; 13 GemVax and Kael Co., Ltd, Seongnam 13461, Korea; 14 Department of Biomedical Sciences, Inha University School of Medicine, Incheon 22332, Korea; 15 Department of Statistics, Inha University, Incheon 22212, Korea

**Keywords:** Alzheimer’s disease, mild cognitive impairment, telomere, progression, amyloid

## Abstract

We investigated whether telomere length (TL) reflecting physical rather than chronological aging is associated with disease progression in the different cognitive stages of Alzheimer’s disease (AD). Study participants included 89 subjects with amyloid pathology (A+), determined through amyloid PET or cerebrospinal fluid analysis, including 26 cognitively unimpaired (CU A+) individuals, 28 subjects with mild cognitive impairment (MCI A+), and 35 subjects with AD dementia (ADD A+). As controls, 104 CU A- individuals were selected. The participants were evaluated annually over two years from baseline. Compared to the highest TL quartile group of MCI A+ participants, the lowest TL quartile group yielded 2-year differences of -9.438 (95% confidence interval [CI] = -14.567 ~ -4.309), -26.708 (-41.576 ~ -11.839), 3.198 (1.323 ~ 5.056), and 2.549 (0.527 ~ 4.571) on the Mini-Mental State Examination, Consortium to Establish a Registry for AD, Clinical Dementia Rating-Sum of Boxes, and Blessed Dementia Scale-Activities of Daily Living, respectively. With this group, the lowest TL quartile group had a significantly greater probability of progressing to ADD than the highest TL quartile group (hazard ratio = 13.16, 95% CI = 1.11 ~ 156.61). Telomere shortening may be associated with rapid cognitive decline and conversion to dementia in MCI A+.

## INTRODUCTION

The number of people with age-related dementia, particularly Alzheimer's disease dementia (ADD), is rapidly increasing due to worldwide aging populations. Over the past decade, significant advances have been made in identifying AD biomarkers, mainly through neuroimaging and cerebrospinal fluid (CSF) molecular analysis. The biomarkers associated with amyloid β (Aβ) plaques include cortical amyloid positron emission tomography (PET) ligand binding [1–3] or low CSF Aβ42 [4], both of which provide evidence of the AD pathophysiological process in vivo. Recent studies using AD biomarkers suggest a 20- to 30-year interval between initial amyloid positivity and the onset of dementia [5]. Approximately 25% of cognitively unimpaired (CU) individuals and 50-70% of individuals with mild cognitive impairment (MCI) in the AD syndromal cognitive staging show amyloid deposition on PET or CSF studies [6–8]. CU individuals with a positive amyloid biomarker are classified as having preclinical AD [9].

Despite extensive research in the field of AD, no treatment has yet been developed to modify its progression. Thus, alternative therapies, including nonpharmacological approaches and risk factor controls, are becoming more important. Interventions targeting diverse risk factors, including more childhood education, exercise, maintaining social engagement, reducing smoking, and management of diabetes mellitus (DM), depression, poor sleep, and midlife hearing loss, obesity, and hypertension might have the potential to delay or prevent a third of dementia cases [10, 11]. Physical activity (PA) has also been associated with slower progression of dementia and with a lower risk of mortality in ADD [12]. However, age is a robust nonmodifiable risk factor for AD [10].

Telomeres are repetitive nucleotide sequences at the end of chromosomes that are shortened by cell division and oxidative stress [13, 14]. Critically short telomeres induce cellular senescence [13, 15], and telomere shortening is associated with aging and age-related chronic diseases [16]. Measurement of leukocyte telomere length (TL) is widely used as a marker of TL in other tissues because of their easy availability [17]. Regulation of TL is the result of the interplay between multiple environmental and genetic factors. Recently, TL has emerged as a promising biomarker to assess the cumulative influence of psychosocial, environmental, and behavioral factors on complex disease risk. Shorter TL is associated with high levels of perceived stress, major depressive disorder, low educational attainment, physical inactivity, and short sleep duration [18]. Therefore, TL is increasingly being studied as a possible epigenomic marker associated with neurodegenerative diseases such as AD [19]. Since the main risk factors associated with telomere shortening are also related to the pathogenesis of AD, it is speculated that telomere attrition may be associated with the progression of AD.

However, the data from published studies on TL in AD and MCI patients are both scarce and contradictory [17, 20–22], which likely reflects methodological shortcomings (i.e. undetermined amyloid pathology) resulting in inadequate group allocation of study subjects. Therefore, the present study aimed to investigate whether TL shortening was associated with AD progression over two years in participants with amyloid pathology determined by PET or CSF studies, at the different cognitive stages of AD.

## RESULTS

### Demographic and clinical characteristics of study subjects

The study included 104 CU A-, 26 CU A+, 28 MCI A+, and 35 ADD A+ cases. Table 1 shows the demographic and clinical characteristics of participants by AD cognitive stage. The CU A- and CU A+ participants had a significantly lower mean age than the MCI A+ and ADD A+ participants. The ADD A+ patients had significantly lower educational levels and Mini-Mental State Examination (MMSE) scores than the other groups. The prevalence of men was lower in the ADD A+ group than in the other groups. The Consortium to Establish a Registry for AD (CERAD) and Clinical Dementia Rating-Sum of Boxes (CDR-SB) scores differed by cognitive stage, with better performance in the order of CU A+ > MCI A+ > ADD A+. The Logical Memory (LM) delayed recall score was significantly higher in the CU A- and CU A+ groups than in the MCI A+ and ADD A+ groups. The CU A- group had significantly lower standard uptake value ratios (SUVRs) on ^18^F-flutemetamol PET and ^11^C-Pittsburgh compound-B (PiB) PET, lower prevalence of Apolipoprotein E (APOE) ε4 carrier, and higher CSF Aβ42 levels than the other groups. The ADD A+ patients had significantly higher CSF total tau (t-tau), tau phosphorylated at Thr181 (p-tau), t-tau/Aβ42, and p-tau/Aβ42 levels than the other groups. There were no differences between the groups regarding mean cortical thickness and TL (Table 1).

**Table 1:** Demographic and clinical characteristics of the participants by AD cognitive stage.

	**CU A-**	**CU A+**	**MCI A+**	**ADD A+**	P^*^	***P <* 0.05^†^**
N	104	26	28	35		
Age, years	67.1 (7.5)	66.7 (7.0)	74.6 (7.8)	75.1 (7.5)	**<0.001**	b, c, d, e
Male	44 (42.3%)	12 (46.2%)	18 (64.3%)	9 (25.7%)	**0.023**	
Education, years	10.1 (5.3)	10.4 (5.1)	10.1 (3.6)	6.3 (4.0)	**0.001**	c, e, f
MMSE	26.4 (2.8)	26.2 (3.1)	23.1 (4.4)	16.7 (3.8)	**<0.001**	b, c, e, f
CERAD	79.1 (12.4)	76.7 (13.5)	55.3 (13.5)	37.4 (10.6)	**<0.001**	b, c, d, e, f
LM delayed recall	12.2 (7.8)	9.1 (6.4)	2.8 (3.5)	0.5 (1.2)	**<0.001**	b, c, d, e
CDR-SB	0.01 (0.05)	0.02 (0.10)	1.21 (0.93)	4.64 (2.30)	**<0.001**	b, c, d, e, f
Geriatric Depression Scale	8.3 (6.1)	11.3 (8.4)	11.9 (7.8)	11.4 (7.5)	**0.045**	
APOE ε4 carrier	15 (14.4%)	11 (42.3%)	12 (42.9%)	18 (51.4%)	**<0.001**	
FMM composite SUVR^‡^	0.57 (0.03)	0.70 (0.14)	0.74 (0.13)	0.82 (0.13)	**<0.001**	a, b, c, e
PiB composite SUVR^§^	1.08 (0.05)	1.43 (0.22)	1.60 (0.19)	1.70 (0.36)	**<0.001**	a, b, c
CSF Aβ42, pg/ml^¶^	556.0 (55.8)	386.5 (108.8)	308.2 (86.8)	275.7 (79.2)	**<0.001**	a, b, c, e
CSF t-tau, pg/ml^¶^	49.5 (10.5)	59.1 (36.0)	60.0 (23.5)	96.4 (62.3)	**<0.001**	c, e, f
CSF p-tau, pg/ml^¶^	16.6 (4.5)	23.4 (10.8)	27.5 (18.7)	39.4 (23.9)	**<0.001**	b, c, e, f
CSF t-tau/Aβ42^¶^	0.09 (0.02)	0.19 (0.19)	0.21 (0.11)	0.40 (0.35)	**<0.001**	c, e, f
CSF p-tau/Aβ42^¶^	0.03 (0.01)	0.07 (0.05)	0.10 (0.08)	0.16 (0.11)	**<0.001**	b, c, e, f
Cortical thickness, mm	3.10 (0.14)	3.05 (0.17)	3.03 (0.14)	2.95 (0.14)	0.119	
Hippocampal volume, cm^3^	5.14 (0.74)	5.04 (0.70)	4.45 (1.10)	4.45 (0.87)	**0.004**	c
Telomere length, kb	7.83 (2.03)	8.39 (2.43)	7.52 (1.95)	7.84 (2.04)	0.538	

Values are means (standard deviations) or number (%). CU, cognitively unimpaired; MCI, mild cognitive impairment; ADD, Alzheimer’s disease dementia; A−, absence of amyloid pathology determined by normal amyloid PET finding or CSF study; A+, presence of amyloid pathology determined by abnormal amyloid PET finding or CSF study; MMSE, Mini-Mental State Examination; CERAD, Consortium to Establish a Registry for AD; LM, logical memory; CDR-SB, Clinical Dementia Rating-Sum of Boxes; APOE, apolipoprotein E; FMM, flutemetamol; PIB, Pittsburgh compound-B; CSF, cerebrospinal fluid; Aß, amyloid ß; t-tau, total tau; p-tau, tau phosphorylated at Thr181. ^*^Chi-square test for categorical variables, analysis of variance for age and education, analysis of covariance (ANCOVA) for GDS with age, sex, and education level as covariates, and ANCOVA for other continuous variables with age, sex, education level, and GDS as covariates. ^†^Tukey method for age and education and Bonferroni analysis for other continuous variables. a, CU A- vs. CU A+; b, CU A- vs. MCI A+; c, CU A- vs. ADD A+; d, CU A+ vs. MCI A+; e, CU A+ vs. ADD A+; f, MCI A+ vs. ADD A+. ^‡^Measured in 53 CU A-, 20 CU A+, 21 MCI A+, and 22 ADD A+ participants. ^§^Measured in 42 CU A-, 6 CU A+, 6 MCI A+, and 10 ADD A+ participants. ^¶^Measured in 69 CU A-, 17 CU A+, 16 MCI A+, and 21 ADD A+ participants.

### Associations between telomere length and cognitive function or AD biomarkers

**Figure 1:**
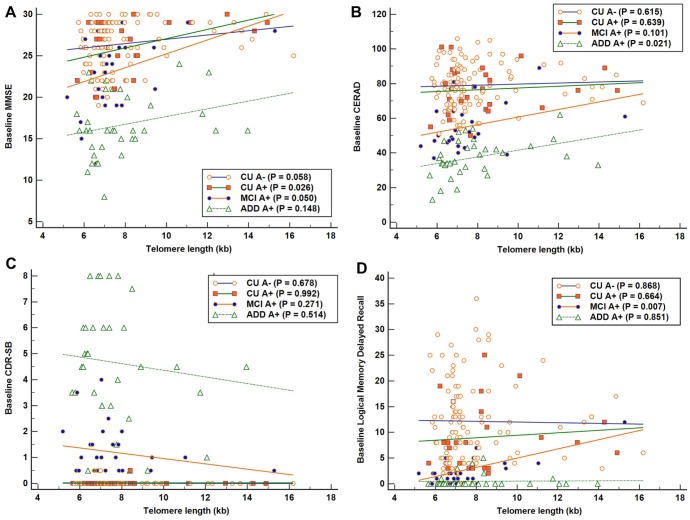
**Associations between telomere length (TL) and baseline cognitive function in each Alzheimer’s disease (AD) cognitive stage group. Simple linear regression was performed with TL as independent variable and each cognitive function test as dependent variable.** (**A**) Significant positive association between TL and Mini-Mental State Examination (MMSE) scores in the cognitively unimpaired (CU) A+ group (R^2^ = 0.190). (**B**) Significant positive association between TL and Consortium to Establish a Registry for AD (CERAD) scores in the AD dementia (ADD) A+ group (R^2^ = 0.152). (**C**) No significant association was detected between TL and Clinical Dementia Rating-Sum of Boxes (CDR-SB) in each AD cognitive stage group. (**D**) Significant positive association between TL and Logical Memory delayed recall scores in the mild cognitive impairment (MCI) A+ group (R^2^ = 0.245). Higher scores suggest better cognition in MMSE, CERAD, and LM delayed recall test, and lower scores suggest better performance in CDR-SB.

The results of simple linear regression analyses of the association between TL as independent variable and each baseline cognitive function test as dependent variable are shown in Figure 1. Across all A+ participants, higher TL was associated with better global cognition measured with MMSE after adjustment for age, sex, and education (Table 2). Higher TLs were associated with better global cognition, measured with MMSE, as well as better memory function, measured with LM delayed recall, in the MCI A+ group. The relationship between CERAD and TL also showed a positive trend in the MCI A+ group, although it did not reach statistical significance.

**Table 2:** Associations between telomere length and cognitive function in the AD cognitive stage groups.

	**MMSE**			**CERAD**			**CDR-SB**			**LM delayed recall**	
	b^*^ (95% CI)	***P^†^***		b^*^ (95% CI)	***P^†^***		b^*^ (95% CI)	***P^†^***		b^*^ (95% CI)	***P^†^***
CU A- (N = 104)											
TL	0.190 (-0.042 ~ 0.422)	0.108		-0.446 (-1.314 ~ 0.421)	0.310		-0.001 (-0.006 ~ 0.004)	0.706		-0.476 (-1.160 ~ 0.208)	0.170
CU A+ (N = 26)											
TL	0.339 (-0.037 ~ 0.715)	0.075		-0.480 (-2.151 ~ 1.192)	0.557		0.002 (-0.016 ~ 0.020)	0.811		-0.268 (-1.218 ~ 0.681)	0.562
MCI A+ (N = 28)											
TL	1.026 (0.047 ~ 2.005)	**0.041**		2.487 (-0.090 ~ 5.060)	0.058		-0.128 (-0.348 ~ 0.092)	0.242		1.048 (0.348 ~ 1.747)	**0.005**
ADD A+ (N = 35)											
TL	0.356 (-0.351 ~ 1.063)	0.312		1.539 (-0.287 ~ 3.365)	0.095		-0.040 (-0.499 ~ 0.420)	0.862		0.072 (-0.162 ~ 0.306)	0.535
All A+ (N = 89)											
TL	0.531 (0.070 ~ 0.991)	**0.024**		1.244 (-0.348 ~ 2.836)	0.124		-0.058 (-0.300 ~ 0.184)	0.636		0.334 (-0.139 ~ 0.808)	0.164

TL, telomere length; CU, cognitively unimpaired; MCI, mild cognitive impairment; ADD, Alzheimer’s disease dementia; A−, absence of amyloid pathology determined by normal amyloid PET finding or CSF study; A+, presence of amyloid pathology determined by abnormal amyloid PET finding or CSF study; MMSE, Mini-Mental State Examination; CERAD, Consortium to Establish a Registry for AD; CDR-SB, Clinical Dementia Rating-Sum of Boxes; LM, logical memory; CI, confidence interval. ^*^Unstandardized coefficient of regression. ^†^Linear regression analysis with each cognitive function test as dependent variable and TL as independent variable, controlling for age, sex, and education.

TL was not correlated with CSF Aβ42, t-tau, p-tau, t-tau/Aβ42, or p-tau/Aβ42 levels, composite SUVRs on ^11^C-PiB PET or on ^18^F-flutemetamol PET, cortical thickness, or hippocampal volume in all A+ participants as well as in each cognitive stage A+ group (Table 3).

**Table 3:** Correlation between telomere length and biomarkers of Alzheimer’s disease in the Alzheimer’s continuum groups.

	**CU A+**		**MCI A+**		**ADD A+**		**All A+**	
	***r***	***P***	***r***	***P***	***r***	***P***	***r***	***P***
CSF Aβ42^*^	0.130	0.608	0.171	0.511	-0.082	0.724	0.104	0.444
CSF t-tau^*^	-0.054	0.830	-0.091	0.737	0.225	0.328	0.082	0.553
CSF p-tau^*^	-0.171	0.498	-0.158	0.545	-0.012	0.958	-0.081	0.554
CSF t-tau/Aβ42^*^	-0.097	0.701	-0.160	0.553	0.355	0.114	0.124	0.366
CSF p-tau/Aβ42^*^	-0.165	0.514	-0.173	0.507	0.133	0.565	-0.027	0.844
FMM composite SUVR^†^	0.008	0.972	0.360	0.109	-0.050	0.824	0.012	0.925
PiB composite SUVR^‡^	-0.535	0.216	0.248	0.591	0.009	0.980	-0.208	0.328
Cortical thickness	-0.122	0.544	-0.006	0.975	-0.060	0.737	-0.039	0.719
Hippocampal volume	0.153	0.447	0.157	0.426	0.022	0.900	0.152	0.156

CU, cognitively unimpaired; MCI, mild cognitive impairment; ADD, Alzheimer’s disease dementia; CSF, cerebrospinal fluid; A−, absence of amyloid pathology determined by normal amyloid PET finding or CSF study; A+, presence of amyloid pathology determined by abnormal amyloid PET finding or CSF study; Aß, amyloid ß; t-tau, total tau; p-tau, tau phosphorylated at Thr181; FMM, flutemetamol; PIB, Pittsburgh compound-B. ^*^Measured in 17 CU A+, 16 MCI A+, and 21 ADD A+ participants. ^†^Measured in 20 CU A+, 21 MCI A+, and 22 ADD A+ participants. ^‡^Measured in 6 CU A+, 6 MCI A+, and 10 ADD A+ participants.

### Influence of telomere length on clinical outcomes over time

There were no differences between the TL quartile groups in each cognitive stage group with regard to age, sex, education, body mass index (BMI), homocysteine level, total sleep time, Mini Nutritional Assessment (MNA) and Geriatric Depression Scale (GDS) scores, or the prevalence of hypertension, DM, dyslipidemia, current smoking, current drinking, sarcopenia, APOE ε4 carriers, or for participants that met the World Health Organization PA guidelines (≥ 600 metabolic equivalent minutes of PA per week) [23] (Supplementary Table 1). Thus, the relationship of the baseline TL quartile level (as the explanatory variable) with the four clinical outcome measures was analyzed using linear mixed models with a function of TL quartile group, age, time, and group x time interaction, in each AD cognitive stage group (Supplementary Table 2).

**Figure 2:**
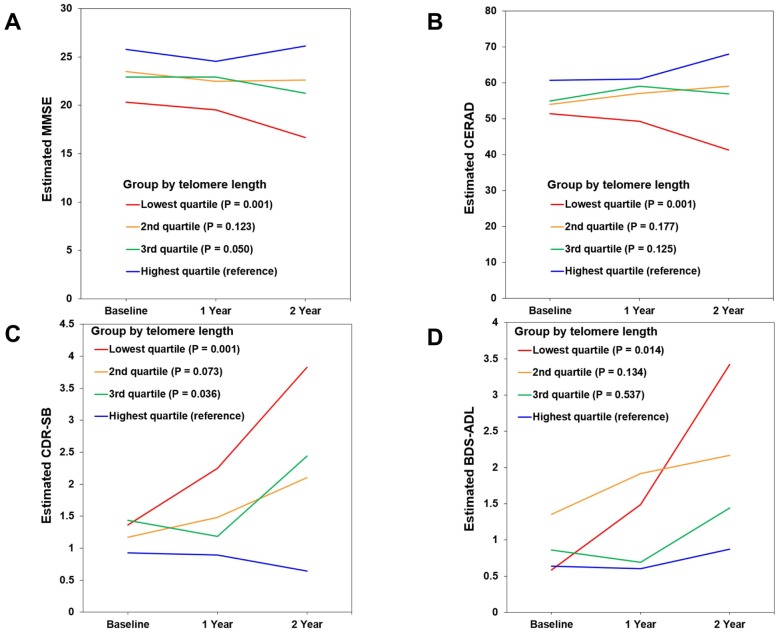
**Changes in cognitive performance over 2 years according to telomere length (TL) in mild cognitive impairment (MCI) A+ participants.** (**A**) Mini-Mental State Examination (MMSE). (**B**) Consortium to Establish a Registry for Alzheimer’s Disease (CERAD). (**C**) Clinical Dementia Rating-Sum of Boxes (CDR-SB). (**D**) Blessed Dementia Scale-Activities of Daily Living (BDS-ADL). Figures show estimated means of clinical outcome measures from baseline to 1- and 2-year follow-up in each TL quartile group. The relationship of baseline TL quartile level (as the explanatory variable) with each clinical outcome measure (as a dependent variable) was analyzed using linear mixed models with a function of TL quartile group, age, time, and group x time interaction. Lower scores suggest worse cognition in MMSE and CERAD, and higher scores suggest worse performance in CDR-SB and BDS-ADL.

In the lowest TL quartile group of MCI A+ participants, the estimated mean differences on 2-year follow-up evaluation compared with the highest TL quartile group were -9.438 (95% confidence interval [CI] = -14.567 ~ -4.309, *P* = 0.001), -26.708 (95% CI = -41.576 ~ -11.839, *P* = 0.001), 3.198 (95% CI = 1.323 ~ 5.056, *P* = 0.001), and 2.549 (95% CI = 0.527 ~ 4.571, *P* = 0.014) on MMSE, CERAD, CDR-SB, and Blessed Dementia Scale-Activities of Daily Living (BDS-ADL), respectively (Figure 2A–2D and Supplementary Table 2). Meanwhile, in the lowest TL quartile group of CU A- participants, the estimated mean difference over the same 2-year period compared with the highest TL quartile group was 0.524 (95% CI = 0.201 ~ 0.846, *P* = 0.002) on the BDS-ADL (Supplementary Table 2).

### Influence of telomere length on AD cognitive stage progression

**Figure 3:**
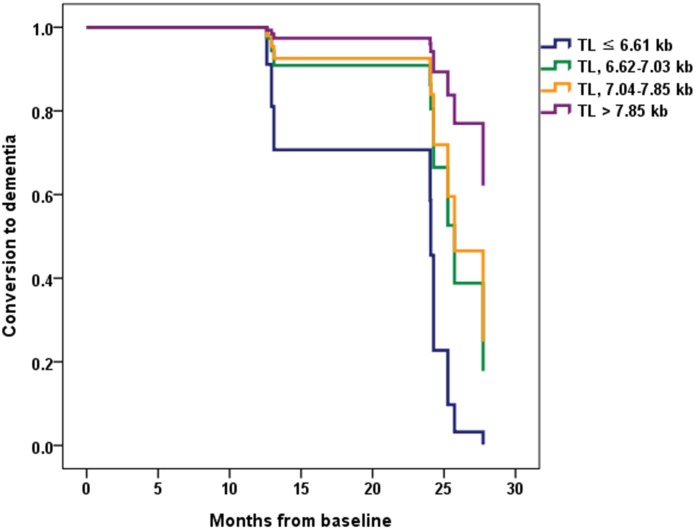
**Conversion from mild cognitive impairment (MCI) to dementia according to the telomere length (TL) quartile groups in MCI A+.** Normalized cumulative conversion data are based on Cox proportional hazards regression analysis adjusted for age as a covariate. The lowest TL quartile group (TL ≤ 6.61 kb) had a significantly greater probability of progressing to dementia compared with the highest TL quartile group (TL > 7.85 kb) in the MCI A+ participants (hazard ratio = 13.16, 95% confidence interval = 1.11 ~ 156.61, *P* = 0.041).

Eight (7.7%) participants from the CU A- group progressed to MCI over two years. Seven (26.9%) participants from the CU A+ group showed cognitive impairment over two years, with five progressing to MCI and two progressing to ADD. Ten (35.7%) participants from the MCI A+ group progressed to ADD within the two-year follow-up. There were no differences between the TL quartile groups in each cognitive stage group with respect to demographic factors, BMI, MNA, homocysteine level, total sleep time, hypertension, DM, dyslipidemia, current smoking, current drinking, sarcopenia, APOE ε4, PA, and GDS (Supplementary Table 1). Therefore, the Cox proportional hazards model was adjusted only for age. This analysis showed that among MCI A+ participants the lowest TL quartile group had a significantly greater probability of progressing to ADD compared with the highest TL quartile group (hazard ratio [HR] = 13.16, 95% CI = 1.11 ~ 156.61, *P* = 0.041) (Table 4 and Figure 3). Among CU A+ participants, rate of progression to MCI or ADD was higher in the lowest TL quartile group than in the other groups. However, for CU A+ participants the HR of conversion to MCI or ADD did not differ significantly between the lowest and highest TL quartile groups.

**Table 4:** Hazard ratios of conversion to MCI or dementia according to the telomere length quartile groups of each CU and MCI groups.

**Group**	**Telomere length (kb)**	**Total**	**Event**	**HR (95% CI)**	P^*^
CU A- (N = 104)	8.11 <	26	2	1 (reference)	
	7.17 – 8.11	26	2	0.84 (0.11 ~ 6.51)	0.866
	6.64 – 7.16	26	3	1.25 (0.20 ~ 8.00)	0.815
	≤ 6.63	26	1	0.51 (0.04 ~ 6.08)	0.597
CU A+ (N = 26)	8.60 <	5	1	1 (reference)	
	7.74 - 8.60	8	2	1.35 (0.11 ~ 16.95)	0.817
	6.68 - 7.73	7	0		0.965
	≤ 6.67	6	4	3.18 (0.32 ~ 31.68)	0.323
MCI A + (N = 28)	7.85 <	7	1	1 (reference)	
	7.04 - 7.85	6	2	2.93 (0.25 ~ 34.93)	0.395
	6.62 - 7.03	8	4	3.63 (0.39 ~ 33.96)	0.259
	≤ 6.61	7	3	13.16 (1.11 ~ 156.61)	**0.041**

CU, cognitively unimpaired; MCI, mild cognitive impairment; A−, absence of amyloid pathology determined by normal amyloid PET finding or CSF study; A+, presence of amyloid pathology determined by abnormal amyloid PET finding or CSF study; HR, hazard ratio; CI, confidence interval. ^*^Cox proportional hazards model adjusted for age.

## DISCUSSION

The present results demonstrate that very short telomeres reflecting pathological aging are associated with a rapid decline in cognitive function in MCI A+ individuals and with conversion from MCI to dementia. In previous long-term follow-up studies, telomere shortening was not associated with either disease progression in MCI or AD [21, 24] or progression of cognitive status in MCI or CU [24]. However, in the referred studies subjects were enrolled based solely on clinical diagnostic criteria, and evaluation of brain amyloid pathology was not described. Thus, some patients without brain amyloid pathology may have been included in the AD or MCI group, while individuals with brain amyloid pathology may have been included in the healthy elderly group. Therefore, the negative results of those studies might be due to misclassification of patients. To our knowledge, this is the first study to evaluate the long-term prognostic effect of TL in patients with amyloid pathology confirmed by AD biomarkers. There was no dose-response relationship between telomere shortening and cognitive decline or between telomere shortening and dementia conversion. This may be because critically short telomeres induce cellular senescence [13, 15].

The long-term prognostic effect of short telomeres was more pronounced in the MCI A+ stage than in the CU A+ or mild ADD A+ stages. There was no relationship between telomere shortening and rapid cognitive decline or ADL deterioration in the ADD A+ group. This may be due to severe neuroinflammation and the relatively weak effect of telomere shortening on the dementia stage of AD. This is supported by a previous CSF study showing that: i) soluble triggering receptor expressed on myeloid cells 2 (sTREM-2), a microglial activation marker, was increased in the CU, MCI, and ADD stages; ii) monocyte chemoattractant protein-1 (MCP-1), a marker of microglial inflammatory reaction, was increased in the MCI and ADD stages but not in the CU stage; and iii) chitinase-3-like protein 1 (CHI3L1), an astroglial activation marker, was increased only in the ADD stage [25]. In the CU A+ stage (preclinical AD), the effect of telomere shortening on clinical deterioration may have been decreased because of the weak neuroinflammatory response and slow progression. In contrast, in the MCI stage of AD, where a moderate neuroinflammatory response occurs, the synergistic effect with telomere shortening may have been strongest. This suggests that the control of risk factors affecting telomere shortening is likely to be more important in the MCI stage. On the other hand, very short telomeres were associated with a rapid decline in ADL performance in the CU A- group. This suggests that pronounced telomere shortening may be causally correlated with functional deterioration in normal aging.

According to cross-sectional analyses, telomere shortening was also associated with poor cognitive function in the MCI stage of AD, as assessed by MMSE and LM tests, and tended to be associated with worse global cognition in the CU stage of AD, as assessed by MMSE. In contrast, telomere shortening was not associated with cognitive function in the ADD stage. These cross-sectional results also suggest that the effect of short telomeres was more pronounced in the MCI stage than in the CU or ADD stages.

In this study, TL was not correlated with AD biomarkers such as CSF Aβ42, t-tau, and p-tau, or composite SUVR on amyloid PET. This suggests that telomere shortening may act as a synergistic factor rather than as a cause of AD pathogenesis. TL was also not correlated with neurodegeneration markers such as cortical thickness or hippocampal volume. However, because this result was obtained from a cross-sectional analysis, a definitive conclusion could not be reached. To clarify this link, it will be necessary to investigate whether telomere length at baseline is related to cortical and hippocampal atrophy progression through follow-up brain imaging analyses.

TL was not significantly different among the A+ cognitive stage groups in our study, nor it varied between each A+ and the CU A- group. This reinforces the notion that, unlike Aβ or tau protein, telomere shortening is not directly related to AD pathogenesis. A previous study reported significantly shorter TL in AD patients than in healthy elderly individuals [20]. However, other studies reported no significant difference in TL between healthy elderly individuals and dementia or MCI patients [24], or in cerebellum TL between pathologically confirmed AD patients and age-matched control subjects [26]. In line with these findings, our results indicate no significant TL shortening in the MCI and ADD stages compared with healthy elderly individuals.

This study has several limitations. First, the number of participants was relatively small, and this may have influenced the lack of association detected between TL and some clinical variables in cross-sectional and longitudinal analyses. Second, the follow-up time (2 years) was short, which may have also prevented detection of true correlations between TL and clinical variables or cognitive status progression that might be revealed over longer periods of time. Third, TL was measured in peripheral leukocytes but not in the brain. Although it might not be a perfect surrogate, the measurement of leukocyte TL is used as a marker of TL in the brain because of the easy availability of blood leukocytes [17]. Indeed, in a previous study leukocyte and cerebellum TLs were directly correlated in individuals with pathologically confirmed sporadic AD [26]. Therefore, in this study leukocyte TL is likely to reflect TL in the brain. Fourth, we evaluated disease progression only through cognitive and functional assessments; we did not investigate the effects of telomere shortening on the progression of brain atrophy through follow-up brain imaging analysis, or on changes in biomarkers through continued amyloid PET imaging or CSF studies. In the future, this analysis may be necessary to increase correlation accuracy and to elucidate the mechanism of action of telomere shortening in AD. Fifth, we did not measure telomerase activity, aberrant telomeric structures, and oxidative stress parameters. Oxidative stress induces a decrease in telomerase activity and telomeric replication defects [27, 28], and reduced telomerase activity as well as telomere attrition are considered markers of accelerated cellular aging [28]. In addition, telomeric replication defects leading to aberrant telomeric structures also contribute to telomere shortening [27]. In the future, the relationship of aberrant telomere structures, telomerase activity, and oxidative stress parameters with cognitive decline should be investigated in individuals with different AD cognitive stages. Sixth, we only evaluated TL at baseline and not since then. There is a possibility that changes in TL during the observation period may have affected the cognitive decline and progression in clinical stage. In the future, the effect of TL changes on disease progression in AD clinical stages should be investigated.

In conclusion, a critically short telomere was associated with a rapid decline in cognitive function in the MCI A+ group and with a rapid conversion from MCI A+ to ADD. TL was not different among the A+ cognitive stage groups and did not differ either between the CU A- group and each A+ clinical stage group. In addition, TL was not correlated with AD biomarkers. This suggests that telomere shortening may act as a synergistic factor rather than as a direct driver of AD pathogenesis. Nevertheless, our data indicate that marked telomere shortening may help predict cognitive decline in AD, especially in the MCI stage.

## MATERIALS AND METHODS

### Participants

In the present study, 89 participants with amyloid pathology (A+; determined by abnormal amyloid PET findings or CSF Aβ42 levels below the cutoff point) and 104 CU individuals without amyloid pathology (A-) as a control group were selected from participants of the independent Validation cohort of the Korean Brain Aging Study for the Early Diagnosis and Prediction of AD (KBASE-V) [7]. The KBASE-V is an independent nationwide cohort from nine hospitals that reconfirms new potential biomarkers identified in the KBASE study and is intended to identify risk and prognostic factors for AD in additional studies. There were 167 CU, 72 MCI, and 56 ADD participants in the KBASE-V. The amyloid PET or CSF study was performed in 131 CU, 58 MCI, and 40 ADD participants. The number of participants with amyloid pathology proven by amyloid PET or CSF studies was 26, 28, and 36 in the CU A+, MCI A+, and ADD A+ groups, respectively. A patient in the ADD A+ group and a participant in the CU A- group were excluded because of inability to measure leukocyte TL. Finally, we included 26 CU A+, 28 MCI A+, 35 ADD A+, and 104 CU A- participants in the present study.

All of the CU participants had normal age-, sex-, and education-adjusted performance on four memory tests of the Korean version of CERAD (word list immediate recall, word list delayed recall, word list recognition, and constructional praxis recall) [29] and had a global CDR scale score of 0 [30]. The participants with MCI met the core clinical criteria for MCI due to AD established by the National Institute on Aging-Alzheimer’s Association (NIA-AA) workgroups [31], and the following criteria modified from the criteria proposed by Petersen et al. [32]: (1) CDR 0.5; (2) memory complaints compared to the participant’s previous memory function by patients, caregivers, or clinicians; (3) a performance score that was lower than 1.5 standard deviations (SDs) below the age-, education-, and sex-adjusted normative means for one or more of the four memory tests included in the CERAD [29]; (4) the ability to perform independent ADL [33]; and (5) not dementia. The ADD participants in the mild dementia stage met the following inclusion criteria: (1) dementia according to the Diagnostic and Statistical Manual of Mental Disorders, 4^th^ Edition (DSM-IV-TR) [34]; (2) probable ADD according to the NIA-AA core clinical criteria [35]; and (3) CDR 0.5 or 1. All participants were aged between 55 and 90 years, and had a reliable informant who could provide the requested information to investigators. For all participants, the exclusion criteria included [7]: (1) the presence of major psychiatric illness; (2) significant neurological or medical condition or comorbidities that could affect cognitive functions; (3) contraindications for magnetic resonance imaging (MRI) scans (e.g., pacemaker, claustrophobia); (4) illiteracy; (5) severe visual or hearing difficulty or serious communication or behavioral problems that could make a clinical examination or brain scan difficult; (6) taking an investigational drug; and (7) pregnancy or breastfeeding.

The study was performed in accordance with the International Harmonization Conference guidelines on Good Clinical Practice, and was approved by the institutional review board of each center. All participants, as well as legal representatives of ADD patients, provided written informed consent prior to study initiation.

### Clinical assessment

All participants underwent physical and neurological examinations and thorough diagnostic procedures including assessment of participants’ cognition, abnormal behaviors, ADL, demographic characteristics, family history, current medications, vascular risk factors, and other comorbidities through the MMSE [29], GDS [36], BDS-ADL [37], and CDR yearly. The participants also underwent the CERAD every year and more detailed neuropsychological tests, including the Wechsler Memory Scale-Fourth edition Korean version LM I, II and recognition test, every two years [7]. Brain MRI and laboratory tests that included blood chemistry; lipid panel; complete blood count; serum levels of folate, vitamin B12, 25-hydroxy vitamin D, and brain-derived neurotrophic factor; C-peptide; glycated hemoglobin (HbA1c); homocysteine; adiponectin; venereal disease research laboratory test; thyroid function test; and APOE genotyping were performed at baseline.

Hypertension was defined as systolic blood pressure ≥ 140 mmHg, diastolic blood pressure ≥ 90 mmHg, or use of antihypertensive medication [38]. DM was defined based on current treatment with insulin or oral hypoglycemic medication, 8-h fasting plasma glucose ≥ 126 mg/dl, or HbA1c ≥ 6.5% [39]. Dyslipidemia was defined as total cholesterol ≥ 200 mg/dl, low-density lipoprotein cholesterol ≥ 130 mg/dl, high-density lipoprotein cholesterol < 40 mg/dl, triglyceride level ≥ 150 mg/dl, or the use of lipid-lowering drugs [40]. Participants’ weight and height were measured while they were wearing light clothing. BMI was calculated as their weight (kg) divided by the square of their height (m^2^). Participants underwent bioelectrical impedance analysis to measure the appendicular skeletal muscle mass index and sarcopenia was diagnosed according to the Asian Working Group criteria [41]. Nutritional status was evaluated using MNA [42]. PA was assessed using the International PA Questionnaire [43], and total sleep time was assessed using the Pittsburgh Sleep Quality Index [44].

### Brain MRI

Brain MRI data were obtained from all participants using a 3.0 T MR scanner, which captured 3D T1-weighted and T2-weighted SPACE sagittal images of 0.8-mm thickness without gap as well as diffusion tensor imaging, axial fluid-attenuated inversion recovery imaging, and resting-state functional MRI. The MRI protocols were based on the AD Neuroimaging Initiative phase 2 MRI protocols [7, 45]. The 3D T1-weighted MRI parameters were as follows: repetition time (TR) = 2300 ms, echo time (TE) = 2.14 ms, inversion time (TI) = 900 ms, Flip Angle (FA) = 9°, and voxel resolution of 0.8 × 0.8 × 0.8 mm^3^ in the Skyra and Trio Tim scanners (Siemens, Washington DC, USA); TR = 7.32 ms, TE = 3.02 ms, TI = 400 ms, FA = 11°, and voxel resolution of 0.8 × 0.8 × 0.8 mm^3^ in the General Electric (GE) Discovery MR750 scanner (GE Healthcare, Milwaukee, WI, USA); and TR = shortest (6.8 ms), TE = shortest (3.1 ms), FA = 9°, and voxel resolution of 0.8 × 0.8 × 0.8 mm^3^ in the Achieva scanner (Philips Healthcare, Andover, MA, USA).

The 3D T1-weighted MRI data were processed using CIVET pipeline v2.1 (http://mcin-cnim.ca/ neuroimagingtechnologies/civet/) [46]. The N3 intensity nonuniformity correction algorithm was used to calibrate the intensity difference due to an inhomogeneity in a magnetic field [47]. Corrected T1-weighted images in native space were aligned to the Montreal Neurological Institute 152 standard space [48]. Non-brain tissue was excluded using the BET algorithm [49]. The registered images were classified into white matter, gray matter, and CSF using an advanced neural-net classifier [46]. The inner surfaces of the cortex were extracted from the partial volume corrected white matter mask using deformable spherical mesh, and then the outer surface of the cortex was automatically extracted using the constrained Laplacian-Based Automated Segmentation with Proximities algorithm [50]. Using the Euclidean distance between the linked vertices of the inner and outer surfaces, cortical thickness values in native space were calculated [51]. After intensity inhomogeneity correction, the corrected T1-weighted images were segmented into the left and right sides of hippocampus using FMRIB’s Integrated Registration and Segmentation Tool [52]. Hippocampal volumes were normalized for total intracranial volume.

### Amyloid PET

Of the total 193 participants in this study, 181 underwent amyloid PET at baseline. There were 116 participants who underwent ^18^F-flutemetamol PET, 64 who underwent ^11^C-PiB PET, and one participant with the historical ^18^F-florbetapir PET result. The PET methods for each of the tracers have been previously described [7]. The SUVR was obtained by using the pons as a reference region on ^18^F-flutemetamol PET and the cerebellar gray matter as the reference region on ^11^C-PiB PET. Composite SUVR values were formed by averaging the SUVR values for frontal, temporal, parietal, occipital, anterior cingulate, and posterior cingulate/precuneus cortices. Based on previous work, elevated Aβ PET was defined as composite SUVR ≥ 0.634 on ^18^F-flutemetamol PET and composite SUVR > 1.21 on ^11^C-PiB PET [7].

### Cerebrospinal fluid analysis

At baseline, 123 of the total 193 participants underwent spinal fluid testing in the morning. CSF was collected in 15-mL polypropylene transfer tubes (Falcon, Corning Science, NY, USA) and immediately centrifuged at 2000 g for 10 min at room temperature (RT). The supernatant (~10 mL) was frozen in dry ice and transferred to Inha University’s laboratory, where CSF biomarkers were measured. After thawing at RT, the shipped CSF samples were mixed with a pipette with a polypropylene tip, and 0.4-mL CSF sample aliquots were frozen in polypropylene tubes (Sarstedt AG and Co., Nümbrecht, Germany) and stored at -80°C until analysis. Aβ42, t-tau and p-tau were measured using the multiplex xMAP Luminex platform with INNO-BIA AlzBio3 kits (Fujirebio Europe, Ghent, Belgium). Based on previous work, participants who underwent CSF studies were deemed to have AD pathology when the CSF Aβ42 was 433.68 pg/ml or lower [7].

### Telomere length assay

Leukocyte TL was examined once at baseline. To this end, DNA was extracted from whole blood using G-DEX^TM^ IIb RBC lysis buffer and G-DEX^TM^ IIb Cell lysis buffer (Intron, MA, USA). DNA hydration was performed with 300 μL of DNA hydration solution (QIAGEN, Hilden, Germany). TL analysis was carried out using a nonradioactive TeloTAGGG TL Assay (Roche Boehringer-Mannheim, Grenzach-Wyhlen, Germany) as described by the manufacturer. Approximately 2-4 μg of DNA from each sample was digested with Hinf I/RsaI enzyme mix and isolated by gel electrophoresis. DNA fragments were transferred to a nylon membrane (Millipore, Bedford, MA, USA) by Southern transfer and hybridized to a digoxigenin (DIG)-labeled probe specific for telomeric repeats. The membrane was incubated with DIG-specific antibodies conjugated to alkaline phosphatase, and the probe was visualized by chemiluminescence using an image analyzer (ImageQuant LAS 4000, GE Healthcare, Little Chalfont, UK). Mean telomeric repeat-binding factor lengths were determined by comparison with molecular weight standards.

### Outcomes

We used 4 summary scores to index disease progression, namely, the MMSE, the CDR-SB, the CERAD, and the BDS-ADL. The MMSE (range 0-30) evaluates the orientation, registration and recall of three words, attention and calculation, language, repetition, and complex commands [29]. The CDR assesses dementia severity along 5 levels of impairment (rated as 0, 0.5, 1, 2, or 3) in each of 6 domains: memory, orientation, judgment and problem solving, community affairs, home and hobbies, and personal care. The CDR-SB score (range 0-18) is the sum of the ratings in each of the 6 domains [30]. The CERAD total score is the sum of the scores of its 7 sub-tests: verbal fluency (animal naming), Boston naming (15 items), word list learning, constructional praxis, word list recall, word list recognition, and constructional praxis recall [29]. The BDS-ADL score (range 0-17) was determined using an 11-item questionnaire assessing activities of daily life, including performing household tasks, coping with small sums of money, remembering short lists of items, finding one’s way indoors, finding one’s way around familiar streets, interpreting surroundings, recalling recent events, tendency to dwell on the past, eating, using the toilet, and dressing [37]. Increases in scores represent worsening on the CDR-SB and BDS-ADL, and improvement on the MMSE and CERAD.

We also investigated cognitive stage transitions (from CU to MCI or dementia, and from MCI to dementia) over two years. The diagnosis of MCI was based on the core clinical criteria for MCI due to AD established by the NIA-AA workgroups [31] and the criteria modified from those proposed by Petersen et al. [32], as mentioned earlier [7]. The diagnosis of dementia was based on DSM-IV-TR criteria for dementia [34]. The diagnosis of ADD was based on the core clinical criteria for probable ADD established by the NIA-AA workgroups [35].

### Statistical analyses

For comparisons among the CU A-, CU A+, MCI A+, and ADD A+ groups, we used chi-square tests for categorical variables and one-way analysis of variance (ANOVA) for age and education level. When statistically significant overall differences were detected in the ANOVA test, pairwise comparisons of means between the diagnosis groups were performed by Tukey’s method. Analysis of covariance (ANCOVA) was performed for statistical analysis of GDS with age, sex, and education level as covariates. ANCOVA was performed for statistical analysis of MMSE, CERAD, LM delayed recall test, CDR-SB, CSF Aβ42, t-tau, p-tau, t-tau/Aβ42, and p-tau/Aβ42 levels, the composite SUVRs on ^11^C-PiB PET and on ^18^F-flutemetamol PET, mean cortical thickness, average score of right and left hippocampal volumes, and TL with age, education level, sex, and GDS as covariates. When an overall statistically significant difference was detected in the ANCOVA test, pairwise comparisons of the means between diagnosis groups were performed by Bonferroni post hoc analysis. Linear regression adjusted for age, sex, and education was used to examine the relationship between TL as independent variable and each baseline cognitive function test as dependent variable in all A+ participants and within each group. Pearson’s correlation coefficients were evaluated to examine the relationship between TL and AD biomarkers in all A+ participants and within each A+ cognitive stage group.

TL was divided into quartiles in each of the CU A-, CU A+, MCI A+, and ADD A+ groups. We used chi-square tests for categorical variables and the Kruskal Wallis test for continuous variables to compare between TL quartile groups the factors associated with TL and AD progression in each AD cognitive stage group. We used linear mixed models with a random subject effect to analyze the relationship between baseline TL quartile level as the explanatory variable and the four clinical outcome measures. The fixed effects included TL quartile group, age, time, and group × time interaction. We used the 1^st^ order autoregressive (AR1) covariance structure in the mixed model, which was selected by the Akaike information criterion (AIC) of the model. The AIC values of AR1 were the lowest in most tested correlation structures (Supplementary Table 3).

To examine the effects of baseline TL on patient’s progression through the different cognitive stages, a Cox proportional hazards model was used after controlling for age with proportional hazard assumption checked by log-log plotting. Survival curves according to TL quartile groups did not intersect and were found to be parallel in each of CU A-, CU A+, and MCI A+ groups. Data are presented as HRs and 95% CIs. Survival was defined as the time between entering the KBASE-V study and the progression of cognitive status (from CU to MCI or dementia, or from MCI to dementia) or a censoring event such as withdrawal from the study or the last completed follow-up examination. Statistical analyses were performed using SPSS 19.0 (SPSS, Chicago, IL, USA). *P* < 0.05 was considered significant.

## Supplementary Material

Supplementary Tables

Supplementary Table 1
