# Supplementary material for: Telomere shortening reflecting physical aging is associated with cognitive decline and dementia conversion in mild cognitive impairment due to Alzheimer’s disease
Source: Aging (Albany NY). 2020 Mar 3;12(5):4407–23. doi: 10.18632/aging.102893 (PMC7093181; doi:10.18632/aging.102893)
Supplement: Supplementary Tables [file aging-12-102893-s002..pdf]

SUPPLEMENTARY TABLES

Please browse Full Text version to see the data of Supplementary Table 1

Supplementary Table 1. Baseline characteristics of the telomere length quartile groups in each AD cognitive stage group.

Supplementary Table 2. Estimated mean differences on 2-year follow-up compared to the highest TL quartile group on clinical outcome measures in each telomere length (TL) quartile group.

TL, kb	MMSE		CERAD		CDR-SB		BDS-ADL	
	Estimate (95% CI)	P^*	Estimate (95% CI)	P^*	Estimate (95% CI)	P^*	Estimate (95% CI)	P^*
CU A-								
8.11 <	0		0		0		0	
7.17 - 8.11	-1.577 (-3.219 ~ 0.066)	0.060	-2.091 (-8.425 ~ 4.243)	0.515	0.072 (-0.042 ~ 0.186)	0.212	0.249 (-0.077 ~ 0.574)	0.134
6.74 - 7.16	-1.208 (-2.835 ~ 0.419)	0.144	-1.567 (-7.800 ~ 4.666)	0.620	-0.046 (0.157 ~ 0.065)	0.419	0.283 (-0.033 ~ 0.599)	0.079
≤ 6.73	-1.185 (-2.829 ~ 0.460)	0.157	-1.412 (-7.761 ~ 4.937)	0.661	-0.027 (-0.140 ~ 0.086)	0.641	0.524 (0.201 ~ 0.846)	0.002
CU A+								
8.60 <	0		0		0		0	
7.74 - 8.60	-3.403 (-7.034 ~ 0.229)	0.066	-15.372 (-31.293 ~ 0.548)	0.058	1.433 (0.257 ~ 2.610)	0.018	-0.125 (-1.719 ~ 1.469)	0.874
6.68 - 7.73	2.176 (-1.655 ~ 6.007)	0.258	3.460 (-13.447 ~ 20.366)	0.670	-0.413 (-1.649 ~ 0.822)	0.504	-1.832 (-3.515 ~ -0.149)	0.034
≤ 6.67	-2.061 (-5.984 ~ 1.861)	0.295	-11.550 (-28.810 ~ 5.709)	0.182	0.042 (-1.227 ~ 1.310)	0.947	-1.161 (-2.884 ~ 0.561)	0.180
MCI A+								
> 7.85	0		0		0		0	
7.04 - 7.85	-4.881 (-9.756 ~ 0.007)	0.050	-11.028 (-25.288 ~ 3.231)	0.125	1.795 (0.126 ~ 3.464)	0.036	0.568 (-1.271 ~ 2.407)	0.537
6.62 - 7.03	-3.509 (-8.014 ~ 0.996)	0.123	-8.929 (-22.096 ~ 4.238)	0.177	1.458 (-0.138 ~ 3.055)	0.073	1.293 (-0.413 ~ 3.000)	0.134
≤ 6.61	-9.438 (-14.567 ~ -4.309)	0.001	-26.708 (-41.576 ~ -11.839)	0.001	3.189 (1.323 ~ 5.056)	0.001	2.549 (0.527 ~ 4.571)	0.014
ADD A+								
> 8.38	0		0		0		0	
7.13 - 8.38	3.278 (-1.749 ~ 8.305)	0.197	4.495 (-7.579 ~ 16.570)	0.458	-0.241 (-4.380 ~ 3.897)	0.907	-1.575 (-4.942 ~ 1.792)	0.353
6.40 - 7.12	0.297 (-4.946 ~ 5.540)	0.910	1.371 (-11.249 ~ 13.991)	0.828	-0.666 (-5.086 ~ 3.753)	0.764	-1.292 (-4.801 ~ 2.217)	0.464
≤ 6.39	-0.385 (-5.267 ~ 4.498)	0.875	-8.630 (-20.370 ~ 3.110)	0.146	0.503 (-3.571 ~ 4.577)	0.806	0.330 (-2.941 ~ 3.600)	0.841

CU, cognitively unimpaired; MCI, mild cognitive impairment; ADD, Alzheimer's disease dementia; A−, absence of amyloid pathology determined by normal amyloid PET finding or CSF study; A+, presence of amyloid pathology determined by abnormal amyloid PET finding or CSF study; MMSE, Mini-Mental State Examination; CERAD, Consortium to Establish a Registry for AD; CDR-SB, Clinical Dementia Rating-Sum of Boxes; BDS-ADL, Blessed Dementia Scale-Activities of Daily Living.
*Linear mixed model with a function of TL quartile group, age, time, and group x time interaction.

Supplementary Table 3. Akaike information criterion values for the tested covariance structures: autoregressive process of order 1 (AR1) and compound symmetry structure corresponding to a constant correlation (CS).

		AR1	CS
CU A-	MMSE	**1280.569**	1284.034
	CERAD	2045.917	**2033.524**
	CDR-SB	**-141.342**	-97.449
	BDA-ADL	**487.704**	491.361
CU A+	MMSE	313.016	**312.117**
	CERAD	469.895	**468.801**
	CDR-SB	**187.429**	191.708
	BDA-ADL	**213.184**	217.940
MCI A+	MMSE	366.924	**355.761**
	CERAD	**499.865**	506.772
	CDR-SB	**250.708**	252.882
	BDA-ADL	**265.504**	270.597
ADD A+	MMSE	**516.503**	518.846
	CERAD	**649.407**	658.293
	CDR-SB	**478.030**	481.643
	BDA-ADL	**462.007**	463.902

CU, cognitively unimpaired; MCI, mild cognitive impairment; ADD, Alzheimer's disease dementia; A−, absence of amyloid pathology determined by normal amyloid PET finding or CSF study; A+, presence of amyloid pathology determined by abnormal amyloid PET finding or CSF study; MMSE, Mini-Mental State Examination; CERAD, Consortium to Establish a Registry for AD; CDR-SB, Clinical Dementia Rating-Sum of Boxes; BDS-ADL, Blessed Dementia Scale-Activities of Daily Living.
