# Supplementary material for: Telomere shortening reflecting physical aging is associated with cognitive decline and dementia conversion in mild cognitive impairment due to Alzheimer’s disease
Source: Aging (Albany NY). 2020 Mar 3;12(5):4407–23. doi: 10.18632/aging.102893 (PMC7093181; doi:10.18632/aging.102893)
Supplement: Supplementary Table 1 [file aging-12-102893-s001..docx]

**Supplementary Table 1. Baseline characteristics of the telomere length quartile groups in each AD cognitive stage group.**

|  |  | CU A- | | | |  |  | CU A+ | | | |  |  | MCI A+ | | | |  |  | ADD A+ | | | |  |
| --- | --- | --- | --- | --- | --- | --- | --- | --- | --- | --- | --- | --- | --- | --- | --- | --- | --- | --- | --- | --- | --- | --- | --- | --- |
| Telomere length, kb | ≤ 6.63 | | 6.64-7.16 | 7.17-8.11 | > 8.11 | | ≤ 6.67 | | 6.68-7.73 | 7.74-8.60 | > 8.60 | | ≤ 6.61 | | 6.62-7.03 | 7.04-7.85 | > 7.86 | | ≤ 6.39 | | 6.40-7.12 | 7.13-8.38 | > 8.38 | |
| N | 26 | | 26 | 26 | 26 | | 6 | | 7 | 8 | 5 | | 7 | | 8 | 6 | 7 | | 10 | | 8 | 9 | 8 | |
| Age, years | 69.4 (9.0) | | 67.5 (6.8) | 66.6 (6.9) | 65.1 (6.9) | | 68.7 (5.3) | | 69.7 (6.1) | 64.4 (8.2) | 64.0 (7.6) | | 78.3 (7.9) | | 71.6 (9.5) | 76.3 (5.4) | 72.7 (6.8) | | 75.5 (8.1) | | 77.9 (6.6) | 75.1 (8.0) | 71.8 (7.2) | |
| Male | 13 (50%) | | 12 (46%) | 10 (38%) | 9 (35%) | | 3 (50%) | | 3 (43%) | 4 (50%) | 2 (40%) | | 3 (43%) | | 5 (63%) | 3 (50%) | 7 (100%) | | 5 (50%) | | 2 (25%) | 1 (11%) | 1 (13%) | |
| Education, years | 10.3 (4.5) | | 9.9 (6.1) | 9.2 (5.7) | 11.1 (4.9) | | 6.8 (6.5) | | 12.4 (4.9) | 10.8 (3.7) | 11.2 (5.0) | | 9.4 (3.6) | | 12.8 (3.2) | 9.0 (3.5) | 8.7 (2.9) | | 7.4 (3.7) | | 4.9 (4.9) | 7.1 (4.3) | 5.5 (3.1) | |
| Hypertension | 13 (50%) | | 8 (31%) | 13 (50%) | 10 (38%) | | 4 (67%) | | 5 (71%) | 5 (63%) | 4 (80%) | | 6 (86%) | | 6 (75%) | 4 (67%) | 2 (29%) | | 3 (30%) | | 3 (38%) | 4 (44%) | 3 (38%) | |
| DM | 1 (4%) | | 8 (31%) | 6 (23%) | 6 (23%) | | 2 (33%) | | 3 (43%) | 1 (13%) | 2 (40%) | | 2 (29%) | | 2 (25%) | 1 (17%) | 2 (29%) | | 0 (0%) | | 2 (24%) | 2 (22%) | 1 (13%) | |
| Dyslipidemia | 22 (85%) | | 18 (69%) | 21 (81%) | 21 (81%) | | 5 (83%) | | 4 (57%) | 7 (88%) | 4 (80%) | | 7 (100%) | | 6 (75%) | 3 (50%) | 5 (71%) | | 6 (60%) | | 4 (50%) | 7 (78%) | 6 (75%) | |
| Current smoking | 1 (4%) | | 1 (4%) | 0 (0%) | 2 (8%) | | 0 (0%) | | 1 (14%) | 0 (0%) | 0 (0%) | | 0 (0%) | | 1 (13%) | 0 (0%) | 1 (14%) | | 0 (0%) | | 0 (0%) | 0 (0%) | 1 (13%) | |
| Current drinking | 6 (23%) | | 12 (46%) | 9 (35%) | 8 (31%) | | 2 (33%) | | 3 (43%) | 3 (38%) | 3 (60%) | | 3 (14%) | | 2 (25%) | 1 (17%) | 3 (43%) | | 0 (0%) | | 2 (25%) | 0 (0%) | 1 (13%) | |
| BMI, kg/m^2^ | 24.3 (2.8) | | 23.8 (2.7) | 24.7 (2.8) | 24.3 (2.6) | | 24.7 (1.9) | | 24.8 (2.9) | 24.8 (2.7) | 26.4 (5.2) | | 25.5 (1.9) | | 24.6 (1.6) | 23.4 (3.0) | 23.6 (2.8) | | 20.8 (2.2) | | 22.1 (2.4) | 21.2 (2.1) | 22.9 (3.8) | |
| Sarcopenia | 3 (12%) | | 2 (8%) | 0 (0%) | 0 (0%) | | 2 (33%) | | 0 (0%) | 0 (0%) | 0 (0%) | | 2 (29%) | | 1 (13%) | 1 (17%) | 0 (0%) | | 5 (50%) | | 2 (25%) | 3 (33%) | 2 (25%) | |
| MNA | 25.4 (2.0) | | 24.4 (3.3) | 25.5 (1.9) | 24.8 (2.2) | | 22.8 (2.7) | | 25.0 (2.5) | 24.3 (2.1) | 23.6 (2.3) | | 24.8 (1.8) | | 24.3 (2.9) | 24.8 (1.3) | 24.0 (1.9) | | 21.1 (3.1) | | 22.3 (2.4) | 20.8 (3.9) | 23.3 (3.6) | |
| Homocysteine, μmol/L | 14.7 (3.3) | | 13.2 (3.2) | 15.2 (5.9) | 13.3 (2.3) | | 12.7 (2.2) | | 17.1 (4.2) | 14.4 (3.5) | 12.7 (2.2) | | 15.8 (4.8) | | 13.8 (2.4) | 14.4 (3.4) | 17.5 (5.2) | | 14.8 (3.8) | | 14.1 (3.0) | 14.7 (7.4) | 13.6 (2.8) | |
| Meet PA guideline^*^ | 22 (85%) | | 21 (81%) | 20 (77%) | 18 (69%) | | 5 (83%) | | 6 (86%) | 7 (88%) | 3 (60%) | | 4 (57%) | | 7 (88%) | 5 (83%) | 7 (100%) | | 6 (60%) | | 6 (75%) | 7 (78%) | 5 (63%) | |
| Total sleep time, hr | 6.8 (1.2) | | 6.5 (1.0) | 6.8 (1.1) | 6.3 (1.3) | | 6.5 (1.2) | | 6.1 (1.5) | 5.8 (2.1) | 6.3 (1.6) | | 6.4 (2.7) | | 7.4 (1.2) | 7.4 (1.7) | 6.1 (1.1) | | 7.9 (2.2) | | 8.3 (1.9) | 6.9 (1.7) | 6.4 (1.5) | |
| APOE ε4 carrier | 3 (12%) | | 4 (15%) | 4 (15%) | 4 (15%) | | 4 (67%) | | 2 (29%) | 4 (50%) | 1 (20%) | | 4 (57%) | | 3 (38%) | 2 (33%) | 3 (43%) | | 3 (30%) | | 4 (50%) | 5 (56%) | 6 (75%) | |
| GDS | 7.2 (4.9) | | 8.9 (6.1) | 9.0 (7.5) | 8.1 (5.7) | | 11.5 (9.6) | | 12.9 (10.1) | 8.9 (4.4) | 12.6 (10.9) | | 14.6 (9.0) | | 13.0 (7.7) | 8.3 (6.5) | 11.0 (8.3) | | 12.5 (6.7) | | 7.6 (5.0) | 12.4 (9.4) | 12.3 (8.2) | |

Values are means (standard deviations) or number (%). CU, cognitively unimpaired; MCI, mild cognitive impairment; ADD, Alzheimer’s disease dementia; A−, absence of amyloid pathology determined by normal amyloid PET finding or CSF study; A+, presence of amyloid pathology determined by abnormal amyloid PET finding or CSF study; DM, Diabetes mellitus; BMI, body mass index; MNA, Mini Nutritional Assessment; PA, physical activity; APOE, apolipoprotein E; GDS, Geriatric Depression Scale. ^*^PA ≥ 600 metabolic equivalents × minutes per week of PA [23]. There were no differences between the telomere length quartile groups in each cognitive stage group with respect to any variables, by Chi-square test for categorical variables and Kruskal Wallis test for continuous variables.
